# Temporal variation in the spectrum and concentration of airborne microalgae and cyanobacteria in the urban environments of inland temperate climate

**DOI:** 10.1007/s11356-023-29341-8

**Published:** 2023-08-18

**Authors:** Matúš Žilka, Mária Tropeková, Eva Zahradníková, Ľubomír Kováčik, Jana Ščevková

**Affiliations:** Department of Botany, Faculty of Natural Sciences, Comenius University, Révová 39, 811 02 Bratislava, Slovakia

**Keywords:** Airborne microalgae, Airborne cyanobacteria, Open-plate cultivation, Hirst-type sampler, Meteorological parameters

## Abstract

Despite their non-negligible representation among the airborne bioparticles and known allergenicity, autotrophic microorganisms—microalgae and cyanobacteria—are not commonly reported or studied by aerobiological monitoring stations due to the challenging identification in their desiccated and fragmented state. Using a gravimetric method with open plates at the same time as Hirst-type volumetric bioparticle sampler, we were able to cultivate the autotrophic microorganisms and use it as a reference for correct retrospective identification of the microalgae and cyanobacteria captured by the volumetric trap. Only in this way, reliable data on their presence in the air of a given area can be obtained and analysed with regard to their temporal variation and environmental factors. We gained these data for an inland temperate region over 3 years (2018, 2020–2021), identifying the microalgal genera *Bracteacoccus*, *Desmococcus*, *Geminella*, *Chlorella*, *Klebsormidium*, and *Stichococcus* (Chlorophyta) and cyanobacterium *Nostoc* in the volumetric trap samples and three more in the cultivated samples. The mean annual concentration recorded over 3 years was 19,182 cells*day/m^3^, with the greatest contribution from the genus *Bracteacoccus* (57%). Unlike some other bioparticles like pollen grains, autotrophic microorganisms were present in the samples over the course of the whole year, with greatest abundance in February and April. The peak daily concentration reached the highest value (1011 cells/m^3^) in 2021, while the mean daily concentration during the three analysed years was 56 cells/m^3^. The analysis of intra-diurnal patterns showed their increased presence in daylight hours, with a peak between 2 and 4 p.m. for most genera, which is especially important due to their potential to trigger allergy symptoms. From the environmental factors, wind speed had a most significant positive association with their concentration, while relative air humidity had a negative influence.

**Supplementary Information:**

The online version contains supplementary material available at 10.1007/s11356-023-29341-8.

## Introduction

The common constituents of atmospheric bioaerosol are fungal spores, pollen grains, microalgae, bacteria, viruses, and fragments of different organisms, e.g., plants, fungi, and animals (Després et al. 2012). Autotrophic microalgae and cyanobacteria, comprising approximately 72,500 species worldwide (Guiry 2012), are ubiquitous, inhabiting both aquatic and terrestrial ecosystems. The common modes of their dissemination in the environment are wind and water currents (Finlay 2002) and under suitable weather conditions they are commonly involved in long-range atmospheric transports up to thousands of kilometres (Lewandowska et al. 2017). Due to their omnipresent occurrence, regular aerosolization, and small size (0.3 to 15 μm in diameter) with appropriate aerodynamic properties (Sharma et al. 2007), their active or dormant stages are also abundant in aerial ecosystems. Their numbers in the lower troposphere reach 10 to 1000 cells/m^3^ (Després et al. 2012).

The autotrophic microorganisms perform numerous positive functions in the atmospheric environment; e.g., they promote cloud formation, thus influencing global hydrological cycle and overall climate on Earth (Tesson and Šantl-Temkiv 2018), and affect the radiation balance of our planet by influencing the albedo of the atmosphere (Després et al. 2012). Despite these positives, they have a potential to harm human health due to production of toxins (Hofbauer 2021) and allergenic molecules (Brunekreef and Forsberg 2005; Genitsaris et al. 2011a) inducing symptoms of allergic respiratory diseases in sensitive individuals (Genitsaris et al. 2011a; Sharma and Rai 2008). These bioparticles are also dangerous in their capability to actively accumulate air pollutants, thus causing more severe medical problems (Touliabah et al. 2022), and their cross-reactivity with other aeroallergens, such as fungal spores (Sharma and Rai 2011). Moreover, the prolonged period for which these minute particles remain suspended in the air raises the probability of exposure to these allergens.

Despite their (i) allergenic potential, (ii) small size, due to which they can penetrate the lower respiratory tract and induce more severe allergic conditions (e.g., allergic asthma) (Facciponte et al. 2018; Lei and Grammer 2019), and (iii) abundant presence in human airways (approximately 1500 microalgae cells per day) (Schlichting 1969), they have received only marginal attention from aerobiologists (Bodkhe Seema 2016; Jadhav and Quazi 2010; Sharma et al. 2006a, 2006b; Sherwood et al. 2020; Singh et al. 2018; Tormo et al. 2001; Wiśniewska et al. 2020, 2022a, 2022b). The main reason is the absence of standard sampling guidelines and the challenging detection and isolation of microalgae and cyanobacteria from air samples. Additionally, most of the studies considering airborne autotrophic microorganisms have been conducted in tropics/subtropics or coastal areas where these hygrophilous organisms are abundant (Sherwood et al. 2014; Singh et al. 2018; Wiśniewska et al. 2022a, 2022b). To our best knowledge, no study related to airborne microalgae and cyanobacteria was performed in the inland temperate climate region so far.

To comprehensively assess the allergenic potential of the air and subsequently implement an appropriate form of prevention or treatment for allergic respiratory diseases, it is necessary to direct our attention not only to commonly investigated bioaerosol components in the air, such as pollen grains and fungal spores but also to previously overlooked constituents, among which microalgae and cyanobacteria undoubtedly belong. Regular continuous monitoring of autotrophic microorganisms in aerobiological samples, especially from densely populated urban areas, is relevant for better understanding of their atmospheric dynamics and thus identifying the potential threat they pose to human health. Therefore, this pilot study from the Central European region aims to identify and quantify airborne microalgae and cyanobacteria in Bratislava city based on 3-year data series and reveal their seasonal, daily, and intra-diurnal patterns. Moreover, the effect of meteorological parameters and air pollutants on their concentrations in the air was appraised. The results of this study can be used in the prevention, diagnosis, and therapy of seasonal respiratory allergies in the study region.

## Materials and methods

### Study area

This study took place in Bratislava (48°10′ N, 17°10′ E), located in the southwestern part of Slovakia. The city is situated between two main geomorphological regions, the Malé Karpaty Mts. (162–559 m a. s. l.) from the north and the Podunajská nížina lowland (200 m a. s. l.) from the south. The Danube River, the second longest river in Europe, flows through the city. Additionally, there are more than 25 natural or artificially created bodies of water in the Bratislava area. These water surfaces, together with various natural (e.g., tree bark, rocks, and soil) and anthropogenic substrates (e.g., roofs and walls), represent potential sources of airborne autotrophic microorganisms. Green spaces comprise approximately one-quarter of the 36,759 ha of Bratislava’s area (Reháčková and Pauditšová 2004), making it one of the greenest cities in Europe. The greenery in the city includes mainly fragments of natural forests and forest parks with natural or semi-natural species. The Malé Karpaty Mts., covered with Carpathian oak-hornbeam forests, reach into the northern part of the city, while fragments of Danubian floodplain forest are situated in the southern part.

Due to its location in Central Europe, Bratislava has a temperate continental climate with warm summers and cold winters. The average annual air temperature is 11.3 °C, with July being the warmest month (21.8 °C) and January being the coldest one (0.8 °C) (2007–2021, Meteorological Observatory Mlynská dolina in Bratislava). The average annual total precipitation is 806 mm, with May being the rainiest (93 mm) and April the driest (43 mm). The average annual wind speed is 2.5 m/s, with prevailing northwest winds.

### Sample collection

Airborne microalgae and cyanobacteria samples were taken using gravimetric open-plate and volumetric Hirst-type method (Hirst 1952). The commonly used Hirst-type aerobiological sampler enables continuous capture of bioparticles from the air and their quantification in a particular air volume. However, identification of autotrophic microorganisms in these samples is ambiguous. The instrument usually captures only single cells or small clumps of dehydrated microalgae and cyanobacteria without the possibility of their cultivation (loss of vitality) or molecular analyses (low concentration in the sample). Their identification, based only on a few cells captured in a single developmental stage, is challenging. A simultaneous use of the gravimetric method with subsequent cultivation on growth medium enables observation of airborne microalgae and cyanobacteria in different developmental stages, leading to their accurate identification. This method does not enable quantification of the microalgal particles in the air, but using both methods, it is possible to get accurate information about both quantity and identity of the airborne autotrophic microorganisms.

The volumetric sampling was performed in 2018, 2020, and 2021 and gravimetric sampling in 2020 and 2021. The volumetric sampler (Burkard Manufacturing Co Ltd.) is placed on the roof of Science Park, Comenius University in Bratislava (48°08′58″N, 17°04′24″ E) at the height of 18 m above ground level. Sterile Petri dishes containing culture medium were placed at the same location once a month for 4 h during days with dry and stable weather (Sharma et al. 2006a; Singh et al. 2018; Wiśniewska et al. 2020). This short, however, effective sampling period prevents the damage of both the cultivation medium and captured microorganisms (Sharma et al. 2007).

The plates were located in an open space without barriers. For each sampling, four Petri dishes of 9-cm diameter, containing Bold basal medium, were used. Exposed dishes were incubated at 24 °C in a cultivation chamber under an 18-W light panel in five fluorescent tubes (cold light) with regular watering to prevent the growth of bacteria and fungi. In each of these gravimetric cultivation (GC) samples, the growth of the microalgal and cyanobacterial colonies was examined daily for 3 to 4 weeks, the length of the observation period depending on the growth rate of the colonies. The cells of every colony were observed under the light and epifluorescence microscopes. A microscopic identification of different stages in the life cycle of individual colonies took place several times during the cultivation period of each dish. The collected microalgae and cyanobacteria were identified to the lowest possible taxonomic level, based on morphology according to different determination keys (Hindák 1978, 2001; Kaštovský et al. 2018a, 2018b; Komárek and Anagnostidis 1999).

The volumetric sampler operates at an airflow rate of 10 l/min. The airborne particles suctioned through a horizontally oriented 2 × 14 mm inlet collide with a rotating drum covered with adhesive tape continuously moving under the inlet at the rate of 2 mm/h. After removal from the sampler (once in 7 days), the exposed tape was cut into segments corresponding to 24-h exposure and placed individually on microscope slides coated with a mixture of gelatin, glycerin, phenol, and distilled water. These semi-permanent slides of volumetric trap (VT) samples were stored until their evaluation. Microalgae and cyanobacteria were counted along 12 vertical transects per slide, each representing 2 h of real-time exposure, under a light microscope, at a magnification of 400 × (Galán et al. 2007). Daily and bi-hourly concentrations of these microorganisms were expressed as the number of cells per cubic meter of air (cells/m^3^).

### Environmental data and statistical analysis

The meteorological parameters used in this study were mean daily values of air temperature (°C), relative humidity (%) and wind speed (m/s), and daily totals of sunshine (hours) and precipitation (mm). The values were measured in the Meteorological Observatory Bratislava – Mlynská dolina (48°09′04″ N, 17°04′14″ E), located close (300 m) to the aerobiological monitoring station. The mean daily concentrations of air pollutants (PM_10_ — particulate matter ≤ 10 μm, O_3_ — ozone, CO — carbon monoxide, and NO_2_ — nitrogen dioxide), expressed in μg/m^3^, were excerpted from the database of the Slovak Hydrometeorological Institute.

The relationships between the mean daily concentration of autotrophic microorganisms and selected environmental variables (mean temperature, relative humidity, wind speed, sunshine, precipitation, PM_10_, O_3_, CO, and NO_2_) were studied by establishing non-parametric Spearman’s correlation coefficients. The analysis was performed for the set of all autotrophic microorganisms as well as for the dominant genera in three time periods: (i) all-year-long, (ii) summer months (June–September), and (iii) non-summer months (January–May, October–December). The distinction between summer and non-summer months was implemented to investigate the impact of individual environmental factors on the concentration of airborne microalgae and cyanobacteria during colder and warmer periods of the year. The data analyses were performed in Statistica 12.

## Results

### Diversity and frequency of airborne microalgae and cyanobacteria from gravimetric samples

In GC samples, we identified seven genera of airborne microalgae in the air of Bratislava in 2020. All of them were classified as members of the green algae (Chlorophyta) taxonomic group, namely *Bracteacoccus* sp., *Desmococcus* sp., *Geminella* sp., *Klebsormidium* sp., *Muriella* sp., *Pseudococcomyxa* sp., and *Stichococcus* sp. Additionally, three genera of Cyanobacteria were observed: *Chroococcus* sp., *Nostoc* sp., and *Phormidium* sp. The same genera were observed in 2021, with the exception of *Klebsormidium* (microalgae) and *Chroococcus* and *Phormidium* (cyanobacteria). The most frequent genus was *Nostoc*, which was observed in 67% of all cultivations, followed by *Bracteacoccus* and *Muriella*, with a prevalence of 60%. Contrarily, the least frequent genera *Desmococcus*, *Klebsormidium*, *Chroococcus*, and *Phormidium* occurred only in 7% of cultivations (Table 1).

**Table 1:** Frequency of airborne microalgae and cyanobacteria in GC (gravimetric cultivation) samples in Bratislava, years 2020–2021

Taxonomic group	Genus	Frequency (%)
Chlorophyta	*Bractaecoccus* sp.	60
	*Desmococcus* sp.	7
	*Geminella* sp.	33
	*Klebsormidium* sp.	7
	*Muriella* sp.	60
	*Pseudococcomyxa* sp.	47
	*Stichococcus* sp.	27
Cyanobacteria	*Chroococcus* sp.	7
	*Nostoc* sp.	67
	*Phormidium* sp.	7

### Sesonal variation in airborne autotrophic microorganism concentrations

Based on the morphological features of the identified cultivated taxa, semi-permanent slides of VT samples were evaluated retrospectively. Six taxa of Chlorophyta were detected, including four coccal algae (*Bracteacoccus* sp., *Chlorella* sp., *Desmococcus* sp., and *Stichococcus* sp.) and two filamentous algae (*Klebsormidium* sp. and *Geminella* sp.) (Table 2; Table S1; Fig. S1). The numbers of other taxa were not significant. They included several cells of both Bacillariophyceae (microalgae) and *Nostoc* (cyanobacteria) (Table 2), as well as a few cells that were impossible to identify and were not included in the results. A total of 57,547 cells of airborne autotrophic microorganisms were identified in Bratislava over 3 years. The annual microalgae and cyanobacteria integral (AMIn, the sum of the mean daily microalgae and cyanobacteria concentrations over the year, Galán et al. 2017) ranged between 15,099 cells*day/m^3^ in 2018 and 24,378 cells*day/m^3^ in 2020 (Table 2). Their proportion in the total annual concentration of all airborne bioparticles (including pollen grains, fungal spores, and fern spores) monitored in the studied area varied between 6.7% in 2021 and 10.5% in 2020 (Table 3).

**Table 2:** Total annual microalgae and cyanobacteria concentrations (cells*day/m^3^) in the air of Bratislava represented in VT (volumetric Hirst-type trap) samples

Taxa/year	2018	2020	2021
Bacillariophyceae	1	4	3
Chlorophyta			
*Bracteacoccus* sp.	7066	15,582	10,225
*Desmococcus* sp.	7105	3469	2544
*Geminella* sp.	21	300	87
*Chlorella* sp.	168	1652	2676
*Klebsormidium* sp.	21	1075	487
*Stichococcus* sp.	713	2284	2041
Cyanobacteria			
*Nostoc* sp.	4	12	7
Total	15,099	24,378	18,070

**Table 3:** Total annual airborne concentrations and percentage contributions of different bioparticles in Bratislava. Data concerning pollen grains, fern spores, and fungal spores were obtained from the aerobiology database of the Department of Botany, Comenius University in Bratislava

Bioparticle type/year	2018		2020		2021	
	Σ	%	Σ	%	Σ	%
Pollen grains (pollen*day/m^3^)	32,262	17.6	60,371	26.1	43,848	16.4
Fungal spores (spores*day/m^3^)	135,428	74.0	146,361	63.3	205,984	76.8
Fern spores (spores*day/m^3^)	124	0.1	155	0.1	201	0.1
Microalgae (cells*day/m^3^)	15,099	8.3	24,378	10.5	18,070	6.7
Total	182,913	100	231,265	100	268,103	100

**Fig. 1:**
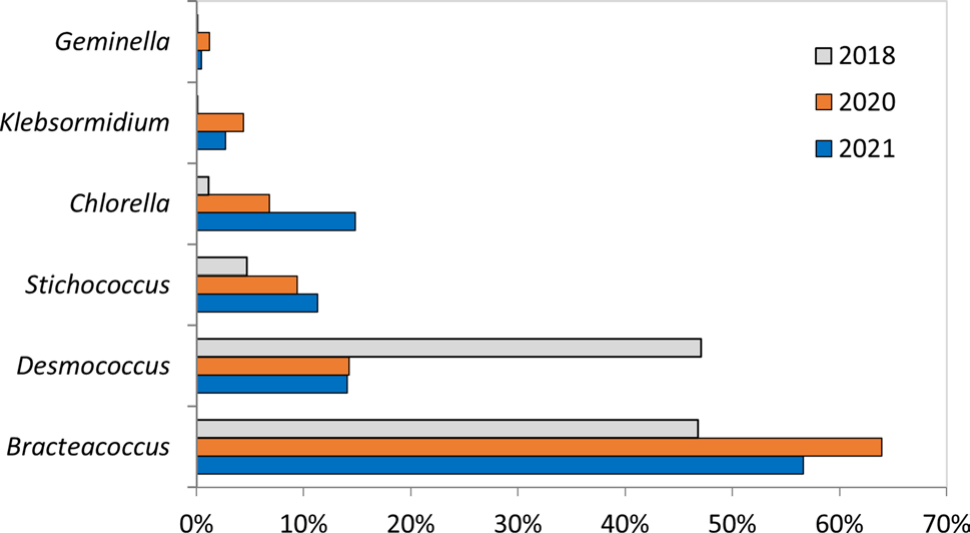
Percentage contribution of airborne microalgae (Chlorophyta) in VT (volumetric Hirst-type trap) samples, in Bratislava, years 2018, 2020–2021

**Fig. 2:**
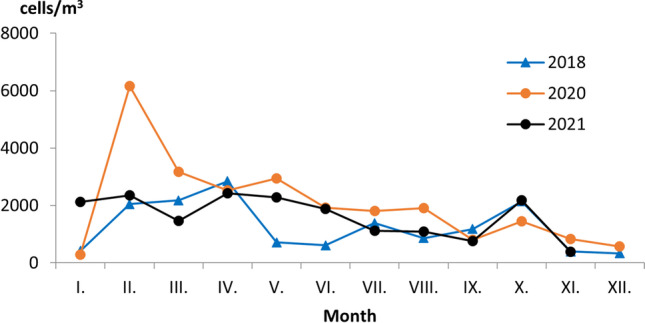
Monthly variation in the total airborne concentrations of microalgae and cyanobacteria in Bratislava, years 2018, 2020–2021

During the analysed years, the maximum contribution was made by *Bracteacoccus*, ranging between 64 and 47% of the AMIn in 2020 and 2018, respectively, while the minimum contribution was made by Bacillariophyceae, *Nostoc*, and *Geminella*, with the mean annual concentration less than 2% of the total (Fig. 1). Microalgae and cyanobacteria were present all year round in the air of the study area, with the highest mean monthly concentration recorded in February (3521 cells/m^3^) and April (2598 cells/m^3^) and lowest in December (450 cells/m^3^) and November (536 cells/m^3^) (Fig. 2).

**Fig. 3:**
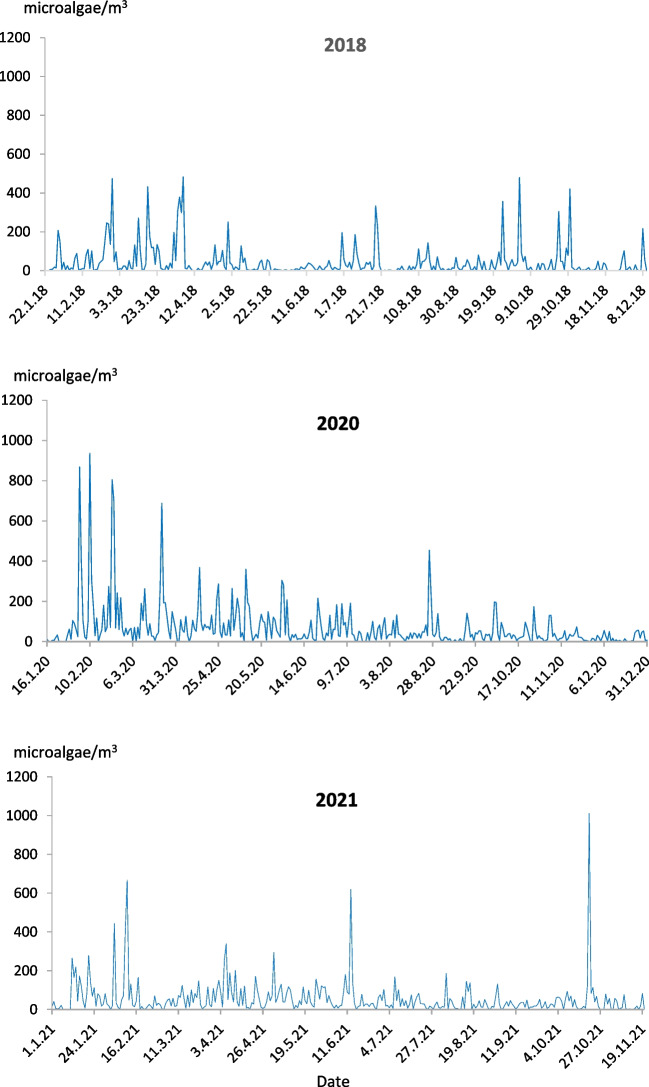
The daily variation in airborne microalgae and cyanobacteria concentrations in Bratislava, years 2018, 2020–2021 (from the first to the last day of aerobiological monitoring in a given year)

The mean daily concentrations of microalgae and cyanobacteria ranged from 44 cells/m^3^ in 2018 to 69 cells/m^3^ in 2020. Considering daily variation in airborne microalgae and cyanobacteria concentrations, our data hint at some seasonal disparities in the peak dates between years (Fig. 3). The peak in 2018 was at the beginning of April, in 2020 at the beginning of February, and in 2021 at the end of October. The highest daily concentrations ranged between 482 and 1011 cells/m^3^ in 2018 and 2021, respectively. In addition to the principal peaks, minor ones were observed at the beginning of October 2018 (497 cells/m^3^) and at the beginning of February 2021 (667 cells/m^3^).

**Fig. 4:**
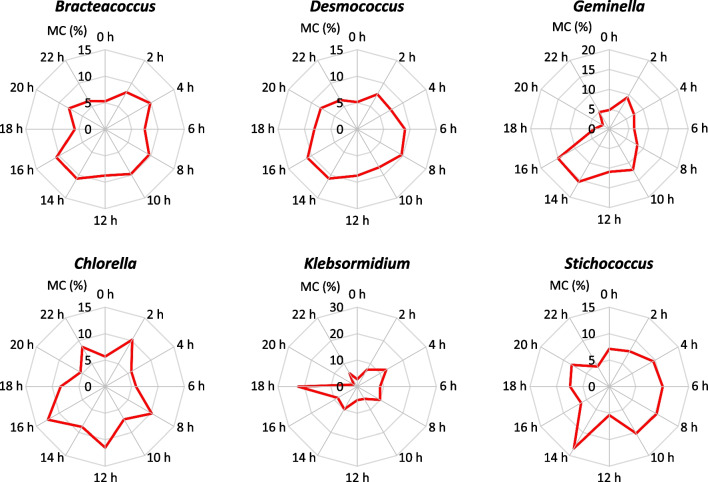
Intra-diurnal variation in the airborne microalgae concentration (MC), expressed in percentage (red lines)

A study of the intra-diurnal periodicity of microalgae concentrations was performed for six abundantly occurring genera in the air of the study area (*Bracteacoccus*, *Desmococcus*, *Geminella*, *Chlorella*, *Klebsormidium*, and *Stichococcus*). In most cases, a relatively constant prevalence of microalgae was observed throughout the day (Fig. 4). However, notable deviations from this trend were found in the cases of *Klebsormidium* and *Geminella*. These taxa were predominantly detected during daylight hours, with airborne microalgae between 8 a.m. and 8 p.m. accounting for 57 and 62% of their overall representation, respectively. Except for *Klebsormidium*, which exhibited its peak concentration at 6 p.m., the remaining taxa demonstrated their highest levels during the afternoon, specifically between 2 and 4 p.m. Similarly, aside from *Bracteacoccus* and *Desmococcus*, which reached their minimum concentrations at midnight, the other taxa registered their lowest levels between 8 and 10 p.m.

### Effect of environmental parameters on airborne microalgae concentration

The weather conditions differed in all years of our study (Table S2). The year 2018 was the warmest, with a mean daily temperature of 12.2 °C, which is 1.4 °C warmer than the long-term average (1983–2021, Meteorological Observatory Bratislava – Mlynská dolina), while 2021, with the mean daily temperature of 10.9 °C, was the coldest. Regarding precipitation levels, all three years received a high but comparable amount of rainfall. With an annual total of 1141 mm, 2020 was the rainiest and received 66% more precipitation than the long-term average. The analysed years also differed in air quality (Table S3). The highest concentration of all air pollutants considered was noted in 2018, while the lowest levels, except for ozone, were observed in 2021.

Spearman’s correlation coefficients between the total daily concentrations of all autotrophic microorganisms detected in airborne samples, alongside the daily concentrations of four most abundant genera (*Bracteacoccus*, *Desmococcus*, *Chlorella*, and *Stichococcus*), and the selected meteorological factors and air pollutants were assessed during three different periods — (i) all-year-long, (ii) non-summer months, and (iii) summer months — throughout the studied years. The results of the correlation analysis are depicted in Table 4. Of all the meteorological parameters assessed, wind speed and relative humidity were the most influential, although less pronounced for *Desmococcus* and *Stichococcus* during the summer months. The relationships with wind speed were significantly positive, while the associations with relative humidity were significantly negative. Precipitation and air temperature had the least impact on the concentration of microalgae in the air. Among the significant associations with air pollutants identified in this study, ozone exhibited a positive correlation with the levels of all microalgae during non-summer and all-year-long periods, except for the all-year-long concentration of *Chlorella*. Conversely, the relationships between other air pollutants (PM_10_, CO, NO_2_) and microalgae were negative. PM_10_ values were significantly associated with all assessed taxonomic groups, although the association was less pronounced in the summer. On the other hand, both CO and NO_2_ exhibited significant associations with *Bracteacoccus* and *Chlorella*. *Desmococcus* was significantly influenced by CO and NO_2_ and *Stichococcus* by CO in non-summer months.

**Table 4:** Spearman’s correlation coefficients between the mean daily concentrations of all airborne autotrophic microorganisms and four most abundant genera and selected weather and air pollution parameters recorded in Bratislava

Genus	Period	N	T	S	RH	P	WS	O_3_	PM_10_	CO	NO_2_
*Bracteacoccus*	AY	968	−0.068*	0.075*	−0.282***	0.01	0.276***	0.13***	−0.232***	−0.287***	−0.256***
	NS	602	0.077	0.188***	−0.385***	0.029	0.239***	0.323***	−0.334***	−0.433***	−0.31***
	S	364	−0.099	0.027	−0.152**	−0.002	0.288***	−0.022	−0.084	−0.286***	−0.227***
*Desmococcus*	AY	968	−0.123***	0.032	−0.224***	0.005	0.205***	0.129***	−0.179***	−0.047	−0.062
	NS	602	0.007	0.192***	−0.368***	0.003	0.195***	0.345***	−0.255***	−0.153***	−0.122**
	S	364	−0.103*	−0.087	−0.017	0.047	0.086	−0.023	−0.122*	−0.065	−0.034
*Chlorella*	AY	968	−0.062	−0.042	−0.148**	0.077	0.176***	−0.003	−0.274***	−0.238***	−0.209***
	NS	602	−0.034	0.048	−0.141***	0.088*	0.17***	0.114**	−0.275***	−0.34***	−0.258***
	S	364	−0.098	0.074	−0.132*	−0.027	0.134*	0.001	−0.104*	−0.185***	−0.132*
*Stichococcus*	AY	968	−0.065	0.049	−0.142***	−0.003	0.17***	0.103*	−0.081*	−0.053	−0.008
	NS	602	0.024	0.165**	−0.266***	−0.34	0.235***	0.263***	−0.144**	−0.195***	−0.072
	S	364	−0.111	−0.036	0.045	0.088	0.043	−0.057	−0.002	0.055	0.066
All AM	AY	968	−0.081*	0.081*	−0.305***	0.033	0.285***	0.15***	−0.279***	−0.29***	−0.242***
	NS	602	0.059	0.21***	−0.421***	0.049	0.259***	0.369***	−0.375***	−0.432***	−0.307***
	S	364	−0.13*	−0.007	−0.146**	0.015	0.268***	−0.059	−0.134	−0.282***	−0.183***

*p < 0.05; **p < 0.01; ***p < 0.001

Statistically significant levels are in bold

*AM*, autotrophic microorganisms; *T*, air temperature; *S*, sunshine hours; *RH*, relative humidity; *P*, precipitation; *WS*, wind speed; *O*_*3*_, ozone; *PM*_*10*_, particulate matter ≤ 10 μm; *CO*, carbon monoxide; *NO*_*2*_, nitrogen dioxide; *N*, number of cases; *AY*, all year long; *NS*, non-summer months: January–May, October–December; *S*, summer months: June–September

## Discussion

### Spectrum and quantity of autotrophic microorganisms from atmospheric samples

Microalgae and cyanobacteria are, similarly to pollen grains and fungal spores, a common constituent of ambient air, although less abundant in aerobiological samples (Essien et al. 2015; Ezikanyi et al. 2018; Tesson et al. 2016). Their airborne concentration depends primarily on resource abundance and meteorological conditions, but can also be influenced by the sampling height. Hirst-type samplers are usually placed 10 to 20 m above ground to capture a mixture of local and long-range transported bioparticles (Lacey and West 2006). However, the number of local bioparticles declines with sampling height due to diluting effect (Rojo et al. 2020). We hypothesize that this effect is more pronounced for autotrophic microorganisms, since their settling velocity is increased by occurrence in clusters or the presence of thick protective layers (Després et al. 2012). Additionally, the cut-point of the Burkard volumetric aerobiological trap is 5.2 l/min (Willeke and Macher 1999), which means that the collection efficiency is weaker for particles smaller than 5.2 μm in diameter. As a result, microalgae and cyanobacteria smaller than 5 μm are significantly under-represented or absent in the VT samples.

Using the volumetric and gravimetric culture-based methods, we observed ten types of microalgae and three types of cyanobacteria in the air of Bratislava, which is less than has been reported from other countries. For instance, Sharma and Singh (2010) and Jadhav and Quazi (2010) noted 33 and 29 genera of autotrophic microorganisms in the atmosphere of Varanasi and Aurangabad (India), respectivelly, while Singh et al. (2018) observed 31 taxonomic units on the Hawaiian Islands. Cyanobacteria were prevalent in all mentioned studies, which, together with the higher diversity of autotrophic microorganisms, is associated with the location of the studied areas in humid tropical climate. Cyanobacteria prefer humid environments (Sahu and Tangutur 2015) while green algae are relatively better adapted to colder and drier conditions (Schlichting 1969); therefore, we anticipated a lower occurrence of Cyanobacteria in the air of Bratislava, which is situated in an inland area of temperate climate zone. This was indeed the case, with the majority of identified genera from the Chlorophyta group.

The spectrum and quantity of genera cultivated from the GC samples may not always coincide with those received from VT samples. For example, based on cultivation, six genera of airborne microalgae and cyanobacteria were noted in Bratislava in November 2020, while *Bractaecoccus* was the only genus detected in the VT samples. On the other hand, in February 2021, *Bractaecoccus*, *Desmococcus*, and *Chlorella* appeared in more than 50% of VT samples although *Muriella* was the only genus in GC samples. These discrepancies are mainly attributed to the challenging cultivation since no medium guarantees the cultivation of all types of microalgae and cyanobacteria. Besides, possible contamination of samples and low viability of some species must be considered (Tesson et al. 2016).

The atmospheric biome, with a number of stress factors such as UV radiation, drought, low temperatures, oxidative stress, and lack of basic nutrients (Radzikowski 2013), is an unnatural and therefore inhospitable environment for microorganisms. These stressors act as a selective force that creates evolutionary pressure on organisms in the atmosphere and largely affects their biodiversity (Fröhlich-Nowoisky et al. 2016). The autotrophic microorganisms present in the air are primarily those that adapted to a life in stress conditions. In this regard, microalgae and cyanobacteria originating from terrestrial rather than aquatic ecosystems are better adapted to atmospheric conditions (Ehresmann and Hatch 1975). Some of the taxa respond by formation of resistant stages, coats, and slime layers (Tesson et al. 2016). Others metabolise various pigments, capable of absorbing solar energy of different wavelengths (Roy et al. 2011). Some genera (e.g., *Coelastrella* or *Scenedesmus*) have multi-layered UV-resistant coats formed by dense cell walls, various antioxidants, and carotenoids (Chiu et al. 2020). In this study, carotenoid accumulation was observed in the genus *Bracteacoccus*, which may largely explain its clear dominance in the samples throughout the studied years. Another abundantly represented genus in the study area, *Chlorella*, is adapted to atmospheric stressors by a tight clustering of cells into a coenobium surrounded by a thick layer of mucilaginous coat (Genitsaris et al. 2011b). The abundance of *Stichococcus* in the samples may have been determined by the small diameter of its cells (Hodac et al. 2016), since the size of bioparticles negatively influences their aerodynamic properties (Finlay 2002). On the other hand, some of the determined taxa were represented in samples only sporadically. For instance, *Bacillariophyceae* have thick silica-encrusted cell walls (Sabater 2009) that increase their weight, so they enter the layer of turbulent atmospheric flow less frequently than other taxa and require higher wind speeds to aerosolize (Marks et al. 2019).

The occurrence of autotrophic microorganisms in the atmosphere is, to some extent, also influenced by the competition between different taxa, as, especially during the summer months, one or only a few dominant genera may “win the battle” for habitat (Sorokin and Dallocchio 2008). *Nostoc*, which inhabits aquatic and terrestrial surfaces and is associated with harmful water blooms, was the most frequently occurring genus in GC samples, but rarely captured in VT samples in our study. The sporadic presence of filamentous cyanobacteria in VT samples can be ascribed to their fragmentation during airborne transportation, resulting in fragments that cannot be identified without prior cultivation (Fröhlich-Nowoisky et al. 2016). In addition, during the vegetation period, VT samples are occupied by other bioparticles such as pollen grains and fungal spores, which can obscure autotrophic microorganisms.

Some genera of airborne microalgae and cyanobacteria recorded during the study period in the air of Bratislava can cause severe allergic conditions in sensitive individuals (Wood and Dietrich 2011). The genera *Chlorella*, *Klebsormidium*, *Bractaecoccus*, and *Phormidium* are known triggers of rhinitis, conjunctivitis, and/or skin irritation, while *Stichococcus* and *Coccomyxa* can also cause asthma attacks (Genitsaris et al. 2011a). The genus *Nostoc* can induce respiratory allergies and is also an active toxin producer (Sharma and Rai 2008). There is insufficient data about the connection of other recorded genera to allergic symptoms and further study is needed in this context.

### Temporal variation in airborne microalgae concentrations

Intra-annual variation in the concentration of microalgae and cyanobacteria in the atmosphere of temperate regions is closely related to seasonal changes in aquatic and terrestrial habitats influencing their release. In winter, freezing conditions on water surfaces hinder the aerosolization of autotrophic microorganisms in aquatic environments, similar to how snow cover affects these organisms in terrestrial environments (Löndahl 2014). However, our study did not find a significant association between temperature and the concentration of airborne microalgae during non-summer months. Although cold winters and mild summers are typical in temperate climates, recent anthropogenic climate change has led to considerably warmer winters and drier summers in Bratislava, resulting in a reduction in snow cover and frozen water bodies (Labudová et al. 2015). This may explain why our findings do not align with the expectation of lower microorganism concentrations in winter. In summer, high temperatures and lack of precipitation can adversely affect the growth of terrestrial microalgae and cyanobacteria. This is partly consistent with the results of our study, as we noted a decline in the concentration of these microorganisms coinciding with rising air temperatures in summer. However, our analysis did not reveal precipitation as an influential weather parameter. In the context of the recent climatic change, we can anticipate a more pronounced decrease in the concentration of airborne autotrophic microorganisms in summer and its further increase during the colder parts of the year.

The diversity and abundance of airborne microalgae and cyanobacteria were roughly homogenous in the study area throughout the analysed years, with a slight increase in their concentration at the end of winter and in spring when a rising temperature and dynamic air circulation promote their drying and aerosolization (Lewandowska et al. 2017). The highest total monthly concentration of autotrophic microorganisms (6152 cells/m^3^) was recorded in February 2020 when the average daily temperature reached 6.2 °C, which is 265% higher than long-term average for this month (1983–2021, Slovak Hydrometeorology Institute), and the wind spead reached 2.7 m/s, which is 1.2 and 0.8 m/s higher than in 2018 and 2021, respectively. Similarly to our results, Tormo et al. (2001) associated an increase in the concentration of microalgae in the atmosphere of the Mediterranean southwestern area of Spain with rising air temperature and wind speed in spring and early summer.

The mean daily concentration of microalgae and cyanobacteria is influenced primarily by the geographical location of the monitoring station, length of monitoring period, as well as the frequency and methods of sampling and sample evaluation; therefore, it varies significantly across studies. Tormo et al. (2001), using the Burkard sampler (daily from February to December), recorded an average daily concentration of airborne autotrophic microorganisms up to 4 cells/m^3^ in south-west Spain. Using a cascade impactor (in average four times per month from January to December), Wiśniewska et al. (2022a) in Northern Poland recorded up to 11 cells/m^3^ from cultivations. In Bratislava, this value reached 56 cells/m^3^ during the three analysed years. Without standardization of the methodic, it is impossible to compare the results of different studies.

Peak daily concentrations of autotrophic microorganisms range from a few hundred to a few thousand cells per cubic meter of air among studies (Genitsaris et al. 2011a; Wiśniewska et al. 2022b), which is in line with the results of our study as 1011 cells/m^3^ is the peak value we noted. However, the ability to accurately record the number of airborne algal cells by some previously used methods, like counting the cells of microalgae and cyanobacteria in precipitation (Dillon et al. 2020; Wiśniewska et al. 2022a), remains questionable.

Regarding the intra-diurnal variation in the airborne microalgae concentration, nearly all recorded genera exhibited peak levels in the afternoon between 2 and 4 p.m. The raised microalgae abundance in the air during hours of increased human outdoor mobility poses a health risk to people sensitive to microalgae-related allergenic molecules. In contrast, a study conducted by Singh et al. (2018) showed high concentrations of Chlorophyta in the Hawaiian Islands during nighttime hours from 6 p.m. to 6 a.m., while cyanobacteria were predominantly present during daytime hours between 6 a.m. and 6 p.m. The different results may be explained by diverse climatic conditions in the tropics and temperate zone. Chlorophyta are abundant in temperate regions, where the climate is colder than in the tropics (Sahu and Tangutur 2015). In the tropics, Chlorophyta are more prevalent during the cooler nights, while during the daytime, they are outnumbered by cyanobacteria which are more adapted to higher temperatures and humidity. Our study found a negative correlation between the concentration of airborne microalgae and air temperature, confirming the preference of Chlorophyta for cooler weather.

### Effect of environmental parameters on airborne microalgae concentration

Statistical analysis showed that the presence of microalgae in the atmosphere is not dependent on a single meteorological variable but on a combination of various environmental parameters, as stated by other researchers (Chiu et al. 2020; Chu et al. 2013; Sharma and Singh 2010; Tormo et al. 2001). In this study, similarly to Tormo et al. (2001), a significant negative correlation was proven between the concentration of airborne microalgae and relative humidity. Although higher air humidity promotes the growth of autotrophic microorganisms (Nakajima et al. 2015), it also leads to their stronger adhesion to surfaces caused by lower evaporation (Yu et al. 2021). Evaporation is associated with the dessication of source microalgae, which facilitates their detachment from the boundary layer to become airborne. Furthermore, high humidity increases the settling velocity of airborne microalgae, since they absorb water from the environment, getting heavier in the process (Sharma et al. 2006b). Wind speed also facilitates detachment of microalgae from the boundary layer (Lewandowska et al. 2017). It had a significant positive influence on the presence of airborne microalgae in our study, as well as in a study conducted by Sharma et al. (2006b). Highest microalgae concentrations were recorded in winter and spring when dynamic air circulation is typical for the study area. Unlike relative humidity, precipitation does not have a significant impact on the concentration of autotrophic microorganisms in the study area. This is due to the diverse effects of rainfall on these microorganisms. It promotes their growth (Gladis-Schmacka et al. 2014; Prieto et al. 2020) but prevents them from becoming airborne or directly removes dispersed microalgae from the air through wash-out mechanisms (Wiśniewska et al. 2022a). In Bratislava, temperature exerts a negative influence on the concentration of airborne microalgae, particularly *Desmococcus*, whereas, in general, the levels of other airborne bioparticles, such as pollen grains and fungal spores, tend to increase as air temperature rises (Grinn-Gofroń and Bosiacka 2015; Schramm et al. 2021). Similar conclusions considering airborne microalgae were reached by other researchers (Singh et al. 2018; Smith 1973). Microalgae are known for their preference for lower temperatures compared to cyanobacteria (Sahu and Tangutur 2015), which agrees with our results and explains their higher abundance in the temperate climatic zone, including the region of Bratislava, as well as their prevalence during the colder period of the year. In contrast to the observed relationship with air temperature, we found a notable positive correlation between the concentration of autotrophic microorganisms in the air and sunshine in non-summer months, except for the genus *Chlorella*. This association can be attributed to sunlight’s stimulating effect on these microorganisms’ growth (Sharma and Singh 2010; Tormo et al. 2001; Wiśniewska et al. 2021).

Regarding air pollution, we noted a significant negative correlation between the concentration of microalgae and CO, NO_2_, and PM_10_. Significant negative correlations were observed between the concentrations of all autotrophic organisms and the levels of CO and NO_2_ throughout all monitoring periods. High levels of CO have been reported to inhibit the growth of source algae (Chen et al. 2018), while elevated nitrate concentrations can rapidly inhibit the growth of cyanobacteria (Wodzinski et al. 1977). Since the amount needed to achieve airborne NO_2_ toxicity is so high that airborne autotrophic microorganisms are unlikely to be affected directly in the atmosphere (Wodzinski and Alexander 1980), we assume that this influence is also exhibited on the source organisms. However, in this context, the negative correlations between the concentration of airborne autotrophic microorganisms and the aforementioned air pollutants can rather be attributed to meteorological parameters that simultaneously influence the concentration of both these air pollutants and microalgae in the study area. Tropospheric ozone exhibited mostly positive associations with the levels of airborne autotrophic microorganisms in the study area, except during the summer months. Its high concentration in the air represents stress for some vascular plants, resulting in increased production of stress hormones, some of which are known allergens (Eckl-Dorna et al. 2010). Similarly, other autotrophic organisms can respond to higher ozone concentrations in the same manner. In this context, the simultaneous increase in the airborne concentration of autotrophic microorganisms and ozone may exacerbate the symptoms of allergic respiratory diseases in urban areas.

## Conclusions

Airborne microalgae and cyanobacteria are a permanent component of bioaerosol, yet poorly studied in many areas of the world despite the risks they pose to susceptible individuals. The findings reported in this publication elucidate the occurrence of ten genera of algae and three genera of cyanobacteria in the air of Bratislava; several of these genera are associated with considerable health risks. Specifically, the prevailing genus identified in the VT samples was *Bracteacoccus*, which, alongside other prevalent genera like *Stichococcus* and *Chlorella*, is a known causative agent responsible for respiratory allergies. Among the genera identified in the GC samples, the prevailing genus was *Nostoc*, which is noteworthy for its dual characteristic of being an allergenic agent as well as an active producer of toxins. Considering that airborne microalgae and cyanobacteria constitute more than 8% of the overall bioaerosol content in the study area, and their daily concentration may exceed 50 cells/m^3^, it can be inferred that these microorganisms have the potential to pose a threat to human health. Furthermore, this risk is intensified by the peak concentrations of microalgae typically occurring during the daytime between 2 and 4 p.m. The findings of our study reveal distinct variations in the dominant species and concentration of microalgae and cyanobacteria in the inland temperate zone compared to coastal areas where they were previously studied. These disparities emphasize the necessity for additional research encompassing diverse biomes to enhance our understanding of the biogeographical distribution of these organisms and the factors that influence their presence in the atmosphere. To effectively assess and mitigate the potential risks posed to sensitive individuals, it is imperative to pursue ongoing research employing a standardized methodology over an extended period.

## Supplementary information

ESM 1 (DOCX 968 kb)

## Data Availability

All data generated or analysed during this study are included in this published article.

## Abbreviations

AMIn: Annual microalgae and cyanobacteria integral
VT: Volumetric trap
GC: Gravimetric sampling and cultivation
