# Supplementary material for: Temporal variation in the spectrum and concentration of airborne microalgae and cyanobacteria in the urban environments of inland temperate climate
Source: Environ Sci Pollut Res Int. 2023 Aug 18;30(43):97616–28. doi: 10.1007/s11356-023-29341-8 (PMC10495494; doi:10.1007/s11356-023-29341-8)

**Temporal variation in the spectrum and concentration of airborne microalgae and cyanobacteria in the urban environments of inland temperate climate**

Matúš Žilka, Mária Tropeková, Eva Zahradníková, Ľubomír Kováčik, Jana Ščevková

**SUPPLEMENTARY INFORMATION:**

**Table S1** Morphological characteristics of the identified genera of autotrophic microorganisms in the air of Bratislava

| Genus | Cell shape | Cell arrangement | Cell size | Mucus layer | Cell wall | Chloroplasts |
| --- | --- | --- | --- | --- | --- | --- |
| *Bracteacoccus* | coccal | tight clusters of 10+ | 9-20 µm | thin or none | thin, often malformed, especially at the edges of the cluster | multiple, without pyrenoids |
| *Chlorella* | coccal | circular clusters of  7-15 | 20-25 µm | thick | thick and distinctive | 1, filling the entire cell, sometimes divided |
| *Chroococcus* | coccal | in pairs or tetrads, amorphous colonies | 3-4,5 µm | thin, colourless | thin, unlaminated | solid, blue-green colour |
| *Desmococcus* | coccal | couples to tetrads grouped in larger clusters | 15(-25) µm | thin | smooth, visually separates cells | 1 without pyrenoid, sometimes reticulate |
| *Geminella* | ellipsoidal | in pairs or short chains | 7-12 µm | thin | thin, create distinct gaps between cells | 1, filling the larger part of the cell |
| *Klebsormidium* | cubic | non-branching long chains | 10 µm (width),  < 250 µm (length) | medium-thick | thick, doubled | deep green colour, adhered to the cell wall |
| *Muriella* | coccal | single cells or diffusion colonies | 4-7 µm | thin | thin | one per cell, cup shaped |
| *Nostoc* | coccal,  filamentous | long chains with heterocytes | 4-6 µm (single cell) | thick | thin, thickened with mucus | filling the entire cell, granular texture |
| *Phormidium* | filamentous | visibly undivided trichomes | 4 µm (width) | none | thin, fine granulated | dark blue-green colour |
| *Pseudococcomyxa* | ellipsoidal,  egg shaped | mucous colonies | 7-12 µm | medium-thick | thin, cells may be asymmetrical | adhered to the cell wall, without pirenoid |
| *Stichococcus* | coccal | larger clumps of 30+ | ~5 µm | none | thin and smooth | 1 per cell, discoid |

**Table S2** Meteorological variables in Bratislava expressed as monthly/yearly averages for temperature, sunshine, relative humidity and wind speed and totals for precipitation.

| Month | T (C) | | |  | S (h) | | |  | RH (%) | | |  | P (mm) | | |  | WS (m/s) | | |
| --- | --- | --- | --- | --- | --- | --- | --- | --- | --- | --- | --- | --- | --- | --- | --- | --- | --- | --- | --- |
|  | 2018 | 2020 | 2021 |  | 2018 | 2020 | 2021 |  | 2018 | 2020 | 2021 |  | 2018 | 2020 | 2021 |  | 2018 | 2020 | 2021 |
| January | 3.3 | 0.4 | 1.4 |  | 1.3 | 2.2 | 1.8 |  | 87 | 90 | 87 |  | 47.7 | 27 | 60.9 |  | 2 | 1.7 | 1.6 |
| February | -0.9 | 6.2 | 2.1 |  | 3.1 | 3.6 | 3.5 |  | 80 | 65 | 84 |  | 56.5 | 58.8 | 39.1 |  | 1.5 | 2.7 | 1.9 |
| March | 3.6 | 7 | 5.5 |  | 3.4 | 6 | 5.8 |  | 74 | 58 | 67 |  | 74.2 | 67.9 | 7.0 |  | 2.1 | 2.1 | 1.6 |
| April | 15.8 | 13 | 8.5 |  | 8 | 9 | 6.3 |  | 62 | 44 | 67 |  | 46.9 | 3.9 | 95.3 |  | 2.3 | 1.6 | 2 |
| May | 18.8 | 13.9 | 13.3 |  | 8.7 | 7.7 | 6.7 |  | 70 | 63 | 74 |  | 121.5 | 110.9 | 139.8 |  | 1.4 | 1.5 | 1.9 |
| June | 20.5 | 18.9 | 22 |  | 8 | 6.9 | 11 |  | 72 | 75 | 62 |  | 103 | 213.5 | 38.8 |  | 1.3 | 1.5 | 1.3 |
| July | 21.8 | 24.1 | 22.7 |  | 9 | 12.4 | 9.9 |  | 68 | 40 | 64 |  | 136.4 | 42.7 | 80.6 |  | 1.1 | 1 | 1.5 |
| August | 23.5 | 21.5 | 19.3 |  | 8.7 | 6.7 | 6.5 |  | 64 | 70 | 76 |  | 70.7 | 152.9 | 151.8 |  | 1.3 | 1.3 | 1.3 |
| September | 17.4 | 17.2 | 17.1 |  | 7 | 6.6 | 6.4 |  | 74 | 71 | 72 |  | 183.1 | 51.8 | 194.5 |  | 1.3 | 1.4 | 1.1 |
| October | 13.5 | 10.9 | 10.6 |  | 4.9 | 2.9 | 5.4 |  | 73 | 86 | 79 |  | 25.8 | 304.6 | 32.6 |  | 1.9 | 1.6 | 1.6 |
| November | 6.7 | 5 | 5.8 |  | 2.5 | 2.1 | 1.9 |  | 87 | 93 | 91 |  | 60.9 | 1.4 | 87.9 |  | 2 | 1.5 | 1.5 |
| December | 1.9 | 3.1 | 2 |  | 1.1 | 1 | 1.4 |  | 87 | 95 | 93 |  | 161.3 | 90.6 | 82.7 |  | 2.1 | 3.2 | 1.7 |
| Year | 12.2 | 11.5 | 10.9 |  | 5.5 | 5.3 | 5.6 |  | 75 | 73 | 76 |  | 1,088 | 1,141 | 1,011 |  | 1.7 | 1.7 | 1.6 |

*T* ‒ air temperature; *S* ‒ sunshine hours; *RH* ‒ relative humidity; *P* – precipitation; *WS* ‒ wind speed

**Table S3** Air pollution characteristics in Bratislava presented as monthly/yearly averages.

| Month | O_3_ (µg/m^3^) | | |  | PM_10_ (µg/m^3^) | | |  | CO (µg/m^3^) | | |  | NO_2_ (µg/m^3^) | | |
| --- | --- | --- | --- | --- | --- | --- | --- | --- | --- | --- | --- | --- | --- | --- | --- |
|  | 2018 | 2020 | 2021 |  | 2018 | 2020 | 2021 |  | 2018 | 2020 | 2021 |  | 2018 | 2020 | 2021 |
| January | 29.1 | 26 | 32.9 |  | 25.8 | 30.1 | 18.9 |  | 493.6 | 493.1 | 389.6 |  | 27 | 29.3 | 20.2 |
| February | 47.8 | 55.2 | 39.8 |  | 31.7 | 13.5 | 33.7 |  | 642.6 | 283.5 | 450.5 |  | 31.3 | 20.4 | 24.1 |
| March | 61.7 | 64.9 | 56.3 |  | 32.7 | 22.7 | 23.9 |  | 649.7 | 303.7 | 347.4 |  | 29.7 | 20.7 | 22.9 |
| April | 72.9 | 81 | 69.4 |  | 25.4 | 23.7 | 17.7 |  | 491.8 | 288.4 | 274.7 |  | 22.1 | 19.7 | 15.5 |
| May | 82.3 | 75.1 | 67.6 |  | 25.1 | 15.5 | 9.6 |  | 425.4 | 247.2 | 233.4 |  | 21 | 15.1 | 14 |
| June | 83.7 | 65.7 | 87.2 |  | 20.3 | 15 | 21.6 |  | 310.9 | 236.4 | 209.6 |  | 18.3 | 14.8 | 15.4 |
| July | 89.8 | 71.1 | 81.2 |  | 19.9 | 18.1 | 17.6 |  | 234.3 | 233.6 | 201.9 |  | 18.2 | 16.9 | 13.6 |
| August | 86.1 | 70.8 | 67 |  | 22.3 | 19.3 | 11.8 |  | 271.9 | 264.4 | 235.5 |  | 23.5 | 18.7 | 13.8 |
| September | 70.2 | 56.5 | 63 |  | 22 | 21 | 17 |  | 401.4 | 295.7 | 289.9 |  | 26.8 | 21 | 20.4 |
| October | 52 | 34.8 | 45.8 |  | 35.3 | 17.4 | 23.6 |  | 414.6 | 324 | 337.1 |  | 28.1 | 17.6 | 21 |
| November | 25.8 | 22.7 | 28.3 |  | 32.1 | 30.2 | 21.2 |  | 597 | 419.9 | 345.8 |  | 28.3 | 17.2 | 21.3 |
| December | 29.3 | 24 | 29.9 |  | 25 | 20.1 | 16.8 |  | 496.3 | 412.7 | 356.8 |  | 27.1 | 16.4 | 21.4 |
| Year | 61 | 53.9 | 55.8 |  | 26.4 | 20.6 | 19.3 |  | 450.9 | 317.1 | 305.1 |  | 25.1 | 19 | 18.6 |

**Fig. S1** Autotrophic microorganisms detected in the air of Bratislava in GC (gravimetric cultivation) and VT (volumetric Hirst-type trap) samples under a light microscope: *Bracteacoccus* (a), *Chlorella* (b), *Desmococcus* (c), *Stichococcus* (d), *Klebsormidium* (e) and *Geminella* (f)


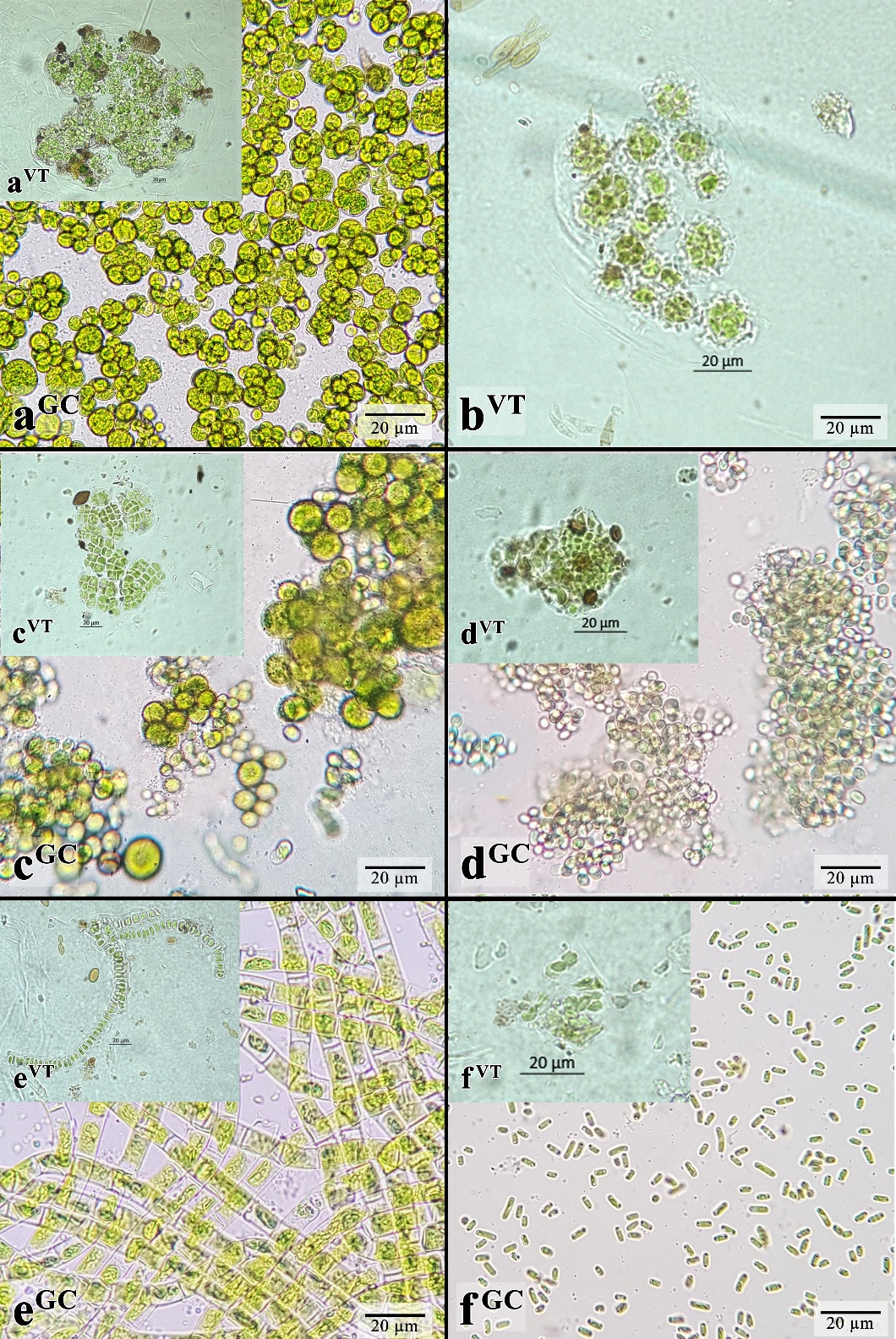

Supplement: Supplementary file 1 — (DOCX 968 kb) [file 11356_2023_29341_MOESM1_ESM.docx]
